# A Novel *KCNA2* Variant in a Patient with Non-Progressive Congenital Ataxia and Epilepsy: Functional Characterization and Sensitivity to 4-Aminopyridine

**DOI:** 10.3390/ijms22189913

**Published:** 2021-09-14

**Authors:** Paola Imbrici, Elena Conte, Rikard Blunck, Fabrizia Stregapede, Antonella Liantonio, Michele Tosi, Maria Cristina D’Adamo, Annamaria De Luca, Vesna Brankovic, Ginevra Zanni

**Affiliations:** 1 Department of Pharmacy—Drug Sciences, University of Bari “Aldo Moro”, 70125 Bari, Italy; elena.conte@uniba.it (E.C.); antonella.liantonio@uniba.it (A.L.); annamaria.deluca@uniba.it (A.D.L.); 2 Department of Physics, Université de Montréal, Montreal, QC H3C 3J7, Canada; rikard.blunck@umontreal.ca; 3 Department of Pharmacology and Physiology, Université de Montréal, Montreal, QC H3C 3J7, Canada; 4 CIRCA, Center for Interdisciplinary Research on Brain and Learning, Université de Montréal, Montreal, QC H3C 3J7, Canada; 5 Unit of Neuromuscular and Neurodegenerative Disorders, Department of Neurosciences, Bambino Gesù, Children’s Hospital, IRCCS, 00146 Rome, Italy; fabrizia.stregapede@opbg.net; 6 Unit of Child Neurology and Psychiatry, University Hospital of Rome Tor Vergata, 00133 Rome, Italy; michele.tosi@opbg.net; 7 Department of Physiology & Biochemistry, Faculty of Medicine, University of Malta, MSD 2080 Msida, Malta; cristina.dadamo@um.edu.mt; 8 Clinic for Child Neurology and Psychiatry, Medical Faculty, University of Belgrade, 11 000 Belgrade, Serbia; vesna.brankovic.npk@gmail.com

**Keywords:** *KCNA2*, ataxia, epilepsy, patch clamp, molecular dynamics, 4-aminopyridine (4-AP)

## Abstract

Kv1.2 channels, encoded by the *KCNA2* gene, are localized in the central and peripheral nervous system, where they regulate neuronal excitability. Recently, heterozygous mutations in *KCNA2* have been associated with a spectrum of symptoms extending from epileptic encephalopathy, intellectual disability, and cerebellar ataxia. Patients are treated with a combination of antiepileptic drugs and 4-aminopyridine (4-AP) has been recently trialed in specific cases. We identified a novel variant in *KCNA2*, E236K, in a Serbian proband with non-progressive congenital ataxia and early onset epilepsy, treated with sodium valproate. To ascertain the pathogenicity of E236K mutation and to verify its sensitivity to 4-AP, we transfected HEK 293 cells with Kv1.2 WT or E236K cDNAs and recorded potassium currents through the whole-cell patch-clamp. In silico analysis supported the electrophysiological data. E236K channels showed voltage-dependent activation shifted towards negative potentials and slower kinetics of deactivation and activation compared with Kv1.2 WT. Heteromeric Kv1.2 WT+E236K channels, resembling the condition of the heterozygous patient, confirmed a mixed gain- and loss-of-function (GoF/LoF) biophysical phenotype. 4-AP inhibited both Kv1.2 and E236K channels with similar potency. Homology modeling studies of mutant channels suggested a reduced interaction between the residue K236 in the S2 segment and the gating charges at S4. Overall, the biophysical phenotype of E236K channels correlates with the mild end of the clinical spectrum reported in patients with GoF/LoF defects. The response to 4-AP corroborates existing evidence that *KCNA2*-disorders could benefit from variant-tailored therapeutic approaches, based on functional studies.

## 1. Introduction

Kv1.2 channels, encoded by the *KCNA2* gene, belong to the Shaker subfamily (Kv1) of voltage-dependent potassium channels, and are widely expressed throughout the central and peripheral nervous system [1,2]. As the other members of the Kv1 family, Kv1.2 channels are tetramers formed by four α-subunits, each composed of six transmembrane segments. The S1-S4 segments form the voltage-sensor domain whereas the S5–S6 form the pore domain and include a membrane reentering P-loop which contains the selectivity filter. Kv1.2 subunits can form homo-tetramers or hetero-tetramers with other members of the Kv1 family, and interact with cytoplasmic auxiliary Kv β-subunits, cytoskeletal scaffolding proteins, and cell adhesion molecules creating diverse channel combinations within neuronal regions [3]. Kv1.2 channels are typically localized together with Kv1.1 along axons and axon terminals, as well as at presynaptic sites, although some neurons express Kv1 proteins in somatic and dendritic compartments [1,4]. Highly density clustering of Kv1.1 and Kv1.2 are found in the juxtaparanodal region surrounding the nodes of Ranvier of mammalian axons, at axonal initial segments and in the pinceau region of cerebellar basket cells, in the hippocampus, cortex, and auditory brainstem [5,6]. Kv1.2 are low-voltage activated slowly inactivating channels that open with small depolarizations close to the resting potential [7,8]. They play an essential role in the initiation and shaping of action potentials, influencing action potential firing patterns, and controlling neuronal excitability.

Deletion or missense mutations of *KCNA2* result in neurologic excitability disorders both in humans and rodents [3,5,8,9]. In mice, the absence of Kv1.2 channels causes a seizure phenotype more severe than Kv1.1 deficiency, possibly due to differences in the function, temporal expression, or localization of the subunits, as well as to the availability of other compensatory subunits [5]. Recently, de novo and inherited heterozygous variants in *KCNA2* gene have been identified in patients presenting with early infantile epileptic encephalopathy, intellectual disability, delayed speech, development delay and ataxia [10,11]. In vitro functional studies provided valuable information to link Kv1.2 variants to the occurrence of ataxia and epilepsy in humans and provide evidence for a significant genotype-phenotype correlation in *KCNA2*-encephalopathy. Missense *KCNA2* variants have been classified into three groups based on the clinical presentations of affected patients and functional defects of mutant channels. They can produce gain-of-function (GoF), loss-of-function (LoF) and even mixed (GoF/LoF) effects [10]. Less severe phenotypes, with focal seizures occurring in early childhood and with more favorable outcome, are associated with LoF variants (such as I263T, R294H, and P405L); more critical infantile epileptic phenotypes with developmental problems, ataxia and cerebellar atrophy are linked to de novo GoF variants (such as R297Q, L298F, E157K); early onset, often uncontrolled, epilepsies, sometimes beginning at neonatal age and followed by severe intellectual disability and ataxia, are associated with variants leading to both GoF and LoF defects (T374A) [10,11,12]

Patients bearing *KCNA2* variants are currently treated with symptomatic antiseizure medications. In some patients, epilepsies only improve with multiple antiepileptic drugs (AEDs) such as lamotrigine, valproic acid, oxcarbazepine and clobazam, whereas in most of them convulsive episodes are severe and pharmacoresistant. Acetazolamide successfully remits ataxia and myoclonic epilepsy caused by *KCNA2* variants in some patients [11,12]. The occurrence of both LoF and GoF defects in *KCNA2*-associated diseases might support the possibility of deploying a variant-tailored therapy for selected patients. At present, however, no approved drug is available, that selectively targets Kv1.2 channels. Recently, 4-aminopyridine (4-AP), a non-specific blocker of Kv1 voltage-gated potassium channels [13], has been reported to improve disease outcome in patients carrying some Kv1.2 GoF variants [14]. This drug is clinically approved for the symptomatic treatment of patients affected by multiple sclerosis and other demyelinated disorders. By blocking overexpressed and mis-localized Kv1 channels, this drug enhances axonal conduction and neuromuscular transmission thus improving ability to walk [15]. 4-AP has also been trialed in some patients affected by episodic ataxia type 2 and downbeat nystagmus where it is supposed to restore Purkinje cells timing and cerebellar output [16].

Here we describe a novel variant in the *KCNA2* gene, the E236K in the S2 segment of the protein, associated with early onset epilepsy and cerebellar ataxia, and test the effect of 4-AP on mutant channels.

## 2. Results

### 2.1. Clinical Description

**Figure 1:**
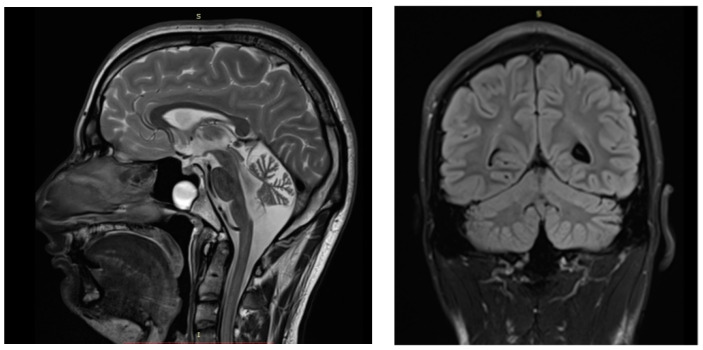
Brain MRI of the patient performed at 23 years. T1-weighted midsagittal section (**left**) and T2-weighted coronal (**right**).

The patient is a 24-year-old man born at term from non-consanguineous healthy parents. Developmental milestones were normal before disease onset at the age of 18 months, with febrile seizures. At the age of 2.5 years, he developed absences, myoclonic and generalized tonic-clonic seizures that were partially controlled with valproate. This drug was later discontinued by the patient. Gait ataxia, dysarthria, and tremors were first observed at the age of 3 years. Brisk tendon reflexes in the lower limbs but no spasticity was present. Language delay, aggressiveness and oppositional behaviour were observed. Moderate intellectual disability, with a global IQ of 56 was established at the age of 6 years. Somatosensory Evoked Potentials (SSEP), visual evoked potential (VEP) and neurography were normal. EEG showed generalized spikes, spike/polyspike-waves of 2 s duration, multifocal asymmetrical spikes, theta activity, and photo paroxysmal response. A brain MRI showed cerebellar atrophy predominant in the vermis (Figure 1) Overall, the patient presented with a clinical diagnosis of syndromic Early Infantile Epileptic Encephalopathy (EIEE) with non-progressive ataxia and intellectual disability. Clinical-functional correlations of individuals harboring *KCNA2* variants compared to the present case are described in Supplementary Table S1 [11].

A novel missense variant in *KCNA2* c.706G > A; p.(E236K) was detected. Segregation analysis demonstrated a de novo origin of the variant, predicted deleterious by in silico tools and not reported in available databases (i.e., dbSNP146, 1000 Genomes, ExAC and GnomAD). We classified the variant as pathogenic, according to ACMG parameters, PS2 PM2 and PP4 [17].

### 2.2. Functional Characterization of Kv1.2E236K Channels

**Figure 2:**
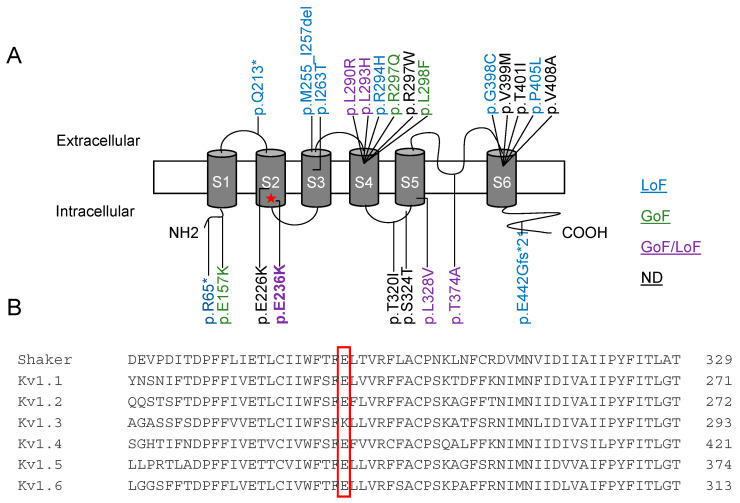
(**A**) Position of identified mutations in the Kv1.2 channel structure and localization of the E236K variant. (**B**) Amino acid alignment of Kv1 channels. LoF, loss-of-function; GoF, gain-of-function; ND, not detected.

The voltage-gated potassium channel Kv1.2 is formed by the tetrameric assembly of four pore-forming alpha subunits, each containing six transmembrane segments (S1–S6). The variant E236K is located in the S2 segment belonging to the voltage sensor domain of Kv channels. This residue is highly conserved within the members of the Kv1 family (Figure 2A,B).

**Figure 3:**
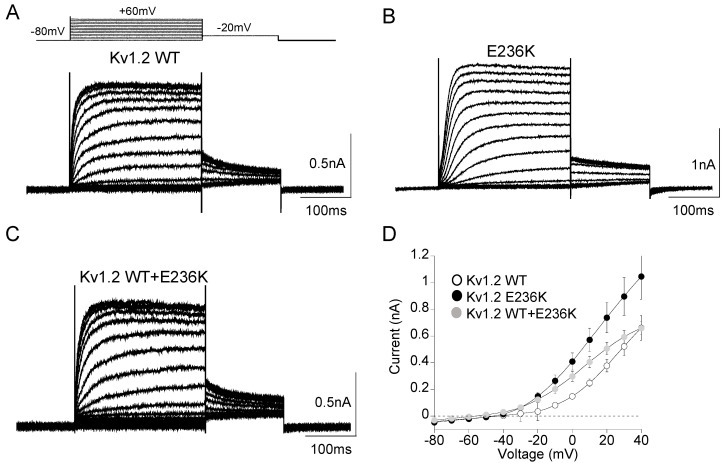
(**A**–**C)** Representative current traces evoked by 200 ms depolarizing steps from a holding potential of −80 to +60 mV from Kv1.2 WT (**A**), E236K (**B**), and Kv1.2 WT+E236K (**C**) channels expressed in HEK293 cells. The voltage protocol is indicated in the upper panel in (**A**). (**D**) Current–voltage relationship for Kv1.2 WT (7 μg), E236K (7 μg), and Kv1.2 WT+E236K (3.5 + 3.5 μg) channels (*n* = 14–36).

As the proband is heterozygous for the disease, he likely possesses heteromeric channels composed by Kv1.2 wild-type (WT) and E236K mutant subunits. To test the hypothesis that the E236K variant altered Kv1.2 function and caused epilepsy and ataxia in the affected patient, we expressed equal amount of WT (7 μg) or E236K (7 μg) cDNAs alone or in 1:1 ratio (3.5 + 3.5 μg) in HEK 293 cells. The current amplitude and biophysical properties of potassium currents elicited by E236K and Kv1.2 WT+E236K channels were then compared with those obtained from WT currents. As shown in Figure 3, E236K currents have similar amplitude to those generated by Kv1.2 WT channels. The co-expression of Kv1.2 WT and E236K subunits gave rise to potassium currents that equals the calculated sum of those carried by WT and mutant channels alone (Figure 3A–D; Table 1).

**Figure 4:**
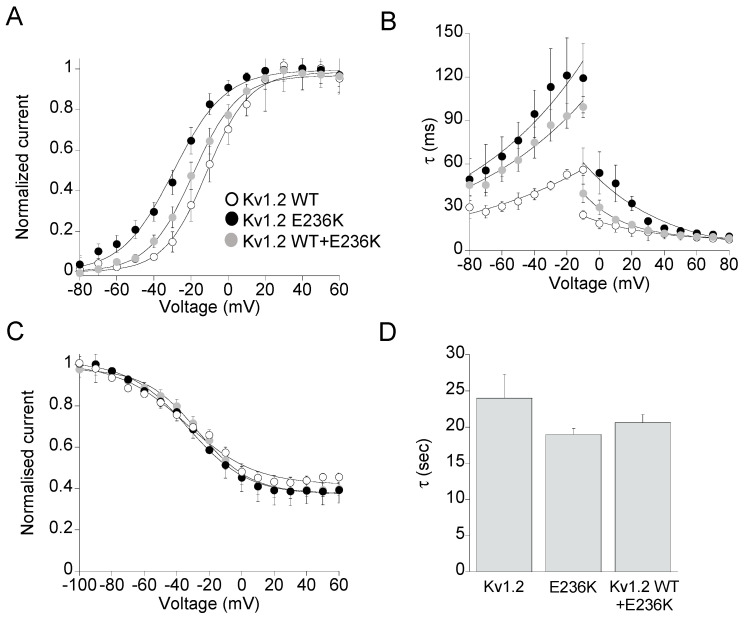
(**A**) The current–voltage relationships for Kv1.2 WT, E236K, and Kv1.2 WT+E236K channels were obtained by plotting the normalized peak tail currents measured at −20 mV as a function of the prepulse potentials and fitting data points with a Boltzmann function (*n* = 10–18 cells). (**B**) Deactivation and activation kinetics measured for Kv1.2 WT, E236K, and Kv1.2 WT+E236K channels. The time constants, resulting from the fit of the activating and deactivating current traces with a single exponential function, were plotted as a function of voltage (*n* = 10–19 cells). (**C**) The voltage-dependence of steady-state inactivation for Kv1.2 WT, E236K, and Kv1.2 WT+E236K channels was obtained by plotting the normalized peak currents measured at +40 mV as a function of the prepulse potentials and fitting data points with a Boltzmann function (*n* = 7–13 cells). (**D**) Bar graphs showing the time constants of the C-type inactivation for the indicated channels calculated by fitting current decay with a single exponential function (*n* = 9–11 cells).

To determine whether the E236K variant may induce modifications of the voltage dependent activation, tail current families were recorded at −20 mV following prepulse commands to several voltages (Figure 4A), and data points were fitted to a Boltzmann function. Mutant E236K channels displayed voltage-dependent activation significantly shifted by 17-mV toward negative potentials compared to Kv1.2 WT, which predicts a GoF effect (Table 1). Potassium currents resulting from the co-transfection of WT and E236K showed voltage-dependent gating that falls between that of WT and E236K homomeric channels, with a 7-mV hyperpolarizing shift of V_1/2_ compared to WT. By contrast, the slope factor k calculated from the Boltzmann fit of tail currents was unaffected by the mutation (Figure 4A, Table 1).

To investigate whether the E283K variant affected the kinetics of activation and deactivation of Kv1.2 channels, the activating and deactivating current traces of either Kv1.2, E236K or Kv1.2 WT+E236K channels were fitted with a single-exponential functions and the calculated time constants at V_1/2_ were plotted as a function of membrane potential (Figure 4B). This analysis revealed that E236K and Kv1.2 WT+E236K channels had 2-fold significantly slower kinetics of deactivation compared with Kv1.2 WT, another GoF effect (Figure 4B, Table 1). Though, the kinetics of activation for E236K channels were much slower than those of Kv1.2 WT at each tested potential, indicating a LoF effect.

Steady state inactivation curves were derived from normalized peak current amplitudes at +40 mV plotted as a function of pre-pulse potentials and fitted to a Boltzmann function. No significant difference was found between the voltage-dependence of the inactivation curves of E236K and Kv1.2 channels (Figure 4C, Table 1). Similarly, the kinetics of slow inactivation were not affected by the E236K variant (Figure 4D).

### 2.3. Effect of 4-Aminopyridine (4-AP) on Kv1.2 WT and E236K Channel

**Figure 5:**
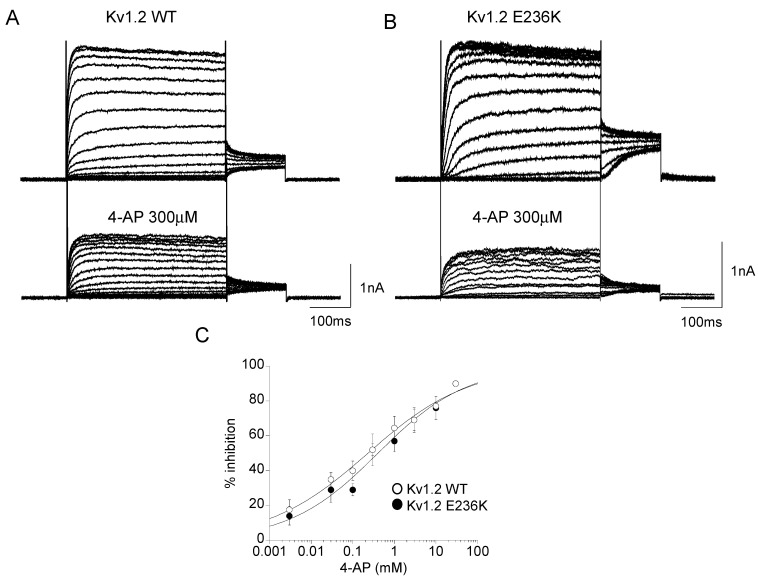
(**A**,**B**) Representative current traces evoked by 200 ms depolarizing steps from a holding potential of −80 to +60 mV from Kv1.2 WT (**A**) and E236K (**B**), before and after the application of 4-aminopyridine (4-AP) 300 μM. (**C**) % of inhibition of potassium current measured at +60 mV as a function of the concentration of 4-AP for Kv1.2 WT and E236K channels. The Hill equation was fitted to the dose-response curves and used to calculate the IC_50_ for each channel type (*n* = 4 cells/dose).

4-AP is a well-known blocker of Kv channels that prevents the final opening step of the channels by binding to the pore cavity [13,18]. It is a basic compound that exists in the protonated or neutral form depending on the pH of the medium. The positively charged protonated form, mimicking a large K^+^ ion, and the amino group suitable for hydrogen bonding, are both necessary to block the channel. To confirm the direct blocking capacity of 4-AP on Kv1.2 channels expressed in HEK 293 cells and to test whether E236K may change 4-AP sensitivity, we recorded whole-cell potassium currents before and after the application of the drug at concentrations ranging from 3 μM to 30 mM at pH = 7 (Figure 5A,B). At each concentration, the relative current was determined as the ratio between the maximal amplitude of the potassium current measured at +60 mV in the absence and in the presence of the drug. The blocking effect was dose-dependent, and the calculated IC_50_ was ~297 ± 5 μM for Kv1.2 channels (Figure 5C). 4-AP blocked E236K potassium currents with an IC_50_ of ~272 ± 9 μM, suggesting that the mutation did not affect the channel sensitivity to 4-AP (Figure 5C).

### 2.4. Molecular Dynamic Simulations

**Figure 6:**
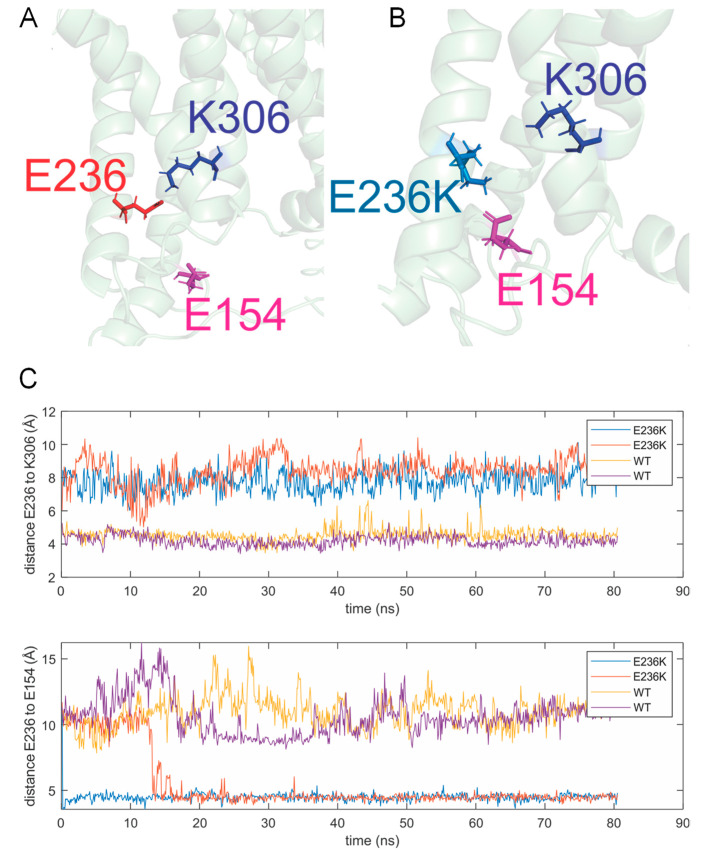
Three-dimensional (3D) protein structure of the Kv1.2 channel shown as a ribbon model and localization of the E236K missense variant. Close-up views of the Kv1.2 WT (**A**) and E236K subunit (**B**). The residues E236, E236K, K306 and E154 are shown in stick representation. Whereas E236 (red) in the WT subunit remains interacting with the 5th gating charge in S4 during the simulation, K306 (blue), the E236K (cyan) does no longer interact with the charges in S4 but rather with E154 (magenta) in the T1-S1 linker close to S1. (**C**) Distances between the Kv1.2 WT or E236K and K306 (S4; upper panel) or E154 (T1-S1; lower panel). The distance to the S4 gating charge is much larger for the mutants, and they flip within the first 15 ns to the E154.

To provide a molecular explanation for the biophysical behavior shown by the E236K channel we first set up molecular dynamic simulations of the Kv1.2 channel composed of two Kv1.2 WT and two adjacent subunits comprising the E236K variant in the open state. In the crystal structure, E236 in the S2 helix points towards a lysine in the voltage sensing S4 (K306, 5th cationic charge in S4; Figure 6A). The coordination between E236 and K306 remained stable during the simulation in the Kv1.2 WT subunits, whereas E236K reoriented very rapidly towards E154 in the T1-S1 linker (Figure 6B). Although the structure of the T1-S1 linker is not well resolved in the available structures, E154 is located proximal the N-terminus of S1, restraining its position by the N-terminus of the S1 helix. Figure 6C shows for the four subunits (two WT and two mutants) the distances between the E236/E236K and K306 (S4; Figure 6C upper panel) and E154 (T1-S1; Figure 6C lower panel). The distance to the S4 gating charge immediately after equilibration is much longer for the mutant subunits E236K (8.5 ± 0.2 Å versus 4.3 ± 0.5 Å for WT). The reorientation of E236K towards E154 occurs at 0.2 and 13.2 ns for the two mutant subunits. The distance reduces from 11.1 ± 0.4 Å to 4.5 ± 0.2 Å, a distance which is typical for salt bridges in the CHARMM (Chemistry at Harvard Macromolecular Mechanics) force field [19].

Intuitively, a missing stabilization of the open state should lead to a voltage-dependence shift to more depolarized potentials. However, one should keep in mind that, according to the current understanding of voltage sensor activation, the gating charges in S4 jump from one negative countercharge in S1–S3 to the next. In this scenario, E236 also stabilizes the cationic S4 gating charges in the closed state, and this interaction would be missing once E236K forms a salt bridge with E154.

A second negative charge (D259 in S3) coordinates K306 in the open state, likely compensating the absence of E236. However, in the closed state, additional positive charges from the S4 cross the hydrophobic “plug” in the voltage sensor and need to be compensated on the cytosolic side of the voltage sensor. In the closed state, with the lack of E154 (due to the interaction with E236K) the surplus of positive charges from the S4 cannot be coordinated and likely explains the stronger destabilization of the closed state and, thereby, the shift of the voltage-dependent activation to more hyperpolarized potentials (GoF). The second anionic charge D259 may help with the transition of the cationic S4 charges, one transferring R303 through the hydrophobic plug, the other coordinating K306. In the presence of a single charge as in E236K, the energy barriers become higher, explaining the slower deactivation kinetics (Figure 4B) and the delay in activation kinetics (Figure 4B).

## 3. Discussion

### 3.1. Genotype-Phenotype Correlation and Mechanistic Hypotheses

Inherited and de novo variants in *KCNA2* have been associated with a spectrum of symptoms ranging from remittent epilepsy to epileptic encephalopathy, development delay, and ataxia. The functional characterization of mutant Kv1.2 channels and the detailed clinical description of carriers allowed to stratify mutations into three subgroups according to the functional defect and to draw a significant genotype-phenotype correlation [11]. Here we describe a novel de novo variant in *KCNA2*, E236K, identified in a Serbian proband affected by non-progressive ataxia, moderate intellectual disability and generalized epilepsy. Kv1.2 E236K channels show a GoF biophysical profile characterized by increased open probability at physiological membrane potentials and slower channel closure. However, the same channels also display slower activation kinetics, a LoF effect. Heteromeric channels recapitulating the condition of the heterozygous carrier show intermediate GoF/LoF behavior that, in agreement with previous studies, correlates with the mild end of the clinical spectrum reported in patients with combined GoF/LoF defects [11].

The E236K variant is located in the S2 segment of the Kv1.2 channel. Despite being outside the hotspots recognized for the other pathogenic *KCNA2* variants (the S4 segment, the pore loop S5–S6 and the S6 residues responsible for channel gating [11], molecular dynamic simulations provide evidence for a stabilizing role of E236 in both the open and the resting state of the channel. Indeed, replacement of the glutamate with a lysine at position 236 results in loss of salt bridge between E236 and the K306 gating charge in S4, which occurs in the WT subunits. Instead, E236K turns towards E154 in the T1-S1 linker and forms a salt bridge here. The net loss of one compensatory charge below the hydrophobic plug, the charge transfer center, in the voltage sensor has likely two consequences; first, the compensatory charges cannot coordinate all cationic gating charges during the transfer leading to higher energy barriers and slower kinetics, and second, in the resting state one compensatory charge is missing, making the channel more susceptible to open at more hyperpolarized potential and explaining the left shift of the voltage dependent activation of the E236K channel.

The cellular mechanisms underlying epilepsy and ataxia following either LoF or GoF variants in *KCNA2* are still unclear. Kv1.2 heteropolymerize with members of the Kv1 family generating complexes with electrophysiological properties set by the subunits type and abundance. Kv1.2-containing channels are expressed at axon initial segment, juxtaparanodal regions and presynaptic terminals of both excitatory and inhibitory neurons where they increase the threshold for neuronal firing and terminate bursts of action potentials, thereby protecting cells from hyperexcitability [8,20,21,22,23,24]. The impact of Kv1.2 variants may be cell-type specific and cause variable phenotypes at different onset ages depending on compensatory mechanisms and still unknown factors [25,26,27].

The cellular basis for the occurrence of epilepsy caused by Kv1.2 LoF variants can be in part supported by the neuronal phenotype of *KCNA2* homozygous knock-out mice. These animals present with severe spontaneous brainstem seizure beginning on postnatal day 15 and die at about day 19 [8]. The increased seizure susceptibility in these mice suggest that Kv1.2 downregulation would likely impair repolarization in excitatory neurons and lower the threshold for AP firing, activating network hyperexcitability. Unexpectedly, however, some brainstem auditory neurons were hypoexcitable in these mice and with larger Kv1 current, suggesting that the absence of Kv1.2 subunits may also result in Kv1.1 predominant complexes that activate at more negative potentials [8]. Regarding GoF variants in potassium channel genes, recent findings support inhibitory neuron-specific mechanisms in mediating the epileptogenic effect. In a mouse model of early-onset seizures carrying a GoF variant in *KCNT1*, a selective functional impairment in cortical inhibitory neurons appeared to be responsible for the epileptogenic potential of the mutation [28]. Likewise, Kv1.2 E236K channels with increased open probability in cortical fast spiking inhibitory interneurons could favor membrane hyperpolarization and silence neuronal firing, leading to reduced GABA release and network disinhibition [10,26]. Alternatively, by ensuring faster membrane repolarization, Kv1.2 GoF may increase Nav channels availability and help sustain high firing rate in excitatory neurons [26,29]. These mechanisms still need to be ascertained.

Cerebellar involvement is one prominent characteristic of *KCNA2* associated disorders. Ataxia was reported in most affected patients, although the degree of severity is more pronounced in the GoF or mixed phenotype group [11], including the one described in this study. The localization and functional role of Kv1.2 channels in the cerebellum can easily account for the ataxic phenotype associated with *KCNA2* defects. Kv1.2/Kv1.1 channels are expressed at the cerebellar basket cells terminals where they regulate GABA release onto Purkinje cells [3]. Pingu mice carrying the LoF *KCNA2* variant I402T, show motor incoordination, myoclonic jerks and tremor due to increased GABA release and reduced cerebellar Purkinje cells output [9]. Although it has not been demonstrated, GoF *KCNA2* variants could as well impact cerebellar functioning, through the mechanisms postulated above. In this regard, some hints can perhaps be drawn from the observation that LoF *CACNA1* variants, impairing Purkinje cell firing, cause ataxia and neurodevelopmental symptoms [30].

It is worth mentioning that in vivo Kv1.2 channels also associate with accessory subunits, interacting proteins and receptors. Kv1.2 channel activity is finely regulated by Kvß subunits, the amino acid transporter Slc7a5, the sigma 1 receptor (Sig-1R), LGI1 and Caspr2, among others, that regulate both trafficking and biophysics [23,31,32,33,34]. The amino acid transporter Slc7a5, for instance, has been reported to silence Kv1.2 activity by reducing channel expression and shifting the voltage dependent activation towards hyperpolarized potential leading to a non-conducting state. Interestingly, epilepsy linked Kv1.2 GoF variants (R297Q and L298F) show increased susceptibility to Slc7a5 modulation in vitro, thus possibly leading to a paradoxical current suppression [32]. The activated sigma 1 receptor, an endoplasmic reticulum protein, favors the transition between slow and fast gating mode upon depolarization and reduces potassium current by binding to the S2-S3 of Kv1.2 channels in HEK cells [23]. LGI-1, a soluble glycoprotein secreted by neurons, sets the density of Kv1 channels at the axon initial segment in the hippocampus and cortex. In a mouse model of autosomal dominant lateral temporal epilepsy (ADLTE), loss of LGI-1 downregulated the expression of Kv1.2 channels thus enhancing neuronal excitability and causing epilepsy [33,34]. The N-terminus of Fragile X Mental Retardation Protein (FMRP) directly binds to a phosphorylated serine motif in the C-terminus of Kv1.2 to regulate excitability of basket cell, GABA release and Purkinje cell firing [35]. The varied regulatory interaction with a number of proteins means that, besides affecting channel biophysical properties, *KCNA2* variants could also impact the physiological modulation and distribution of Kv1.2 channel. In addition, other genetic variants, epigenetics and environmental factors may affect the clinical outcome [36]. Therefore, the outcome of a pathogenic variant as well as patient’s response to drugs may be far more complex than expected by in vitro functional studies. Future studies using neurons derived from patients’ iPSCs are needed to determine the patient-specific mechanisms by which Kv1.2 variants lead to complex epileptic encephalopathy and ataxia [37,38].

### 3.2. Towards Precision Medicine for KCNA2-Disorders

Due to the high variability of clinical outcome following the identification of a genetic variant, the application of precision medicine in ataxia and epilepsy is still difficult [36]. Patients affected by *KCNA2* diseases are treated with a combination of AEDs sometimes with limited benefit [11,12]. Interestingly, fampridine (Fampyra^®^), a modified release form of the Kv channel blocker 4-AP, is being offered, as a more specific therapy, to patients carrying *KCNA2* GoF variants with seizure reduction and improvements in movements and cognition [14,25,29]. As mentioned above, this drug already represents an approved symptomatic treatment to improve motor function in those suffering from multiple sclerosis and other demyelinated disorders and has been trialed in some gait disorders of cerebellar origin [15,16,30,39,40]. Off-label treatment with 4-AP also improved the clinical outcome of one patient affected by vesicle-associated membrane protein 2 (VAMP2)-related epilepsy through increased exocytosis and improved GABAergic tone [41]. In this context, we tested here the effect of 4-AP on both Kv1.2 WT and E236K channels and show that the E236K variant does not modify channel sensitivity to the drug. Thus, 4-AP may represent an interesting potential approach to personalizing the treatment of patients carrying specific *KCNA2* variants. As said, the mechanisms underlying epilepsy and ataxia due to *KCNA2* GoF variants are still unknown, thus mere hypotheses can be drawn to explain the clinical potential of 4-AP. If Kv1.2 GoF variants impair GABAergic tone in inhibitory networks, then 4-AP would prolong action potential duration and increase neurotransmitter release, thus silencing hyperexcitability and improving epilepsy outcome. 4-AP improves cerebellar output and ataxia in some patients affected by episodic ataxia type 2, possibly through a restoration of Purkinje cells firing [16,30]. 4-AP may share a similar mode of action in patients carrying *KCNA2* variants. Of course, any possible approach to evaluate the efficacy of 4-AP therapy on *KCNA2* patients of the GoF or mixed GoF/LoF functional subclass, should consider outcome measures such as Scale for Assessment and Rating of Ataxia (SARA and SARA@home) and gait analysis tools [42,43], and monitoring of seizure frequency through consecutive EEGs and patient-compiled questionnaires. Further studies are needed to reinforce trial readiness and efficacy of 4-AP treatment in *KCNA2* patients, also taking advantage of worldwilde clinical research driven platforms (eg. Ataxia Global Initiative (AGI) https://ataxia-global-initiative.net). The clinical usefulness of this potassium channel blocker may be hampered by its proconvulsant activity and cardiac safety issues at higher doses, thus the benefit/risk profile of 4-AP in *KCNA2* epilepsy should be carefully investigated and eventually new derivatives developed [14,44].

Kv1.2 channels activators directed against LoF mutations would be as well desirable. The upregulation of Kv1.2 channels through docosahexaenoic acid, a non-specific Kv agonist, has been shown to normalize Purkinje cells firing and improve behavioral deficits in a mouse model of Fragile X syndrome [35,45]. Finally, studies using animal models suggest that protective interactions between two ion channels variants (*KCNA1* and *SCN2A* or *CACNA1A*) may modify the phenotypic expression of diseases and highlight additional molecular targets and approaches for drug discovery [46,47].

## 4. Materials and Methods

### 4.1. Clinical Diagnosis

The patient was recruited at the Clinic for Child Neurology and Psychiatry of the University of Belgrade. Blood samples were obtained after written informed consent from all participating subjects. The patient underwent a detailed neurological and neuroradiological examination.

### 4.2. Genetic Testing

The patient was included in a next-generation sequencing (NGS) panel of genes whose mutations are causative of various forms of cerebellar ataxias. Genomic DNA was extracted from peripheral blood of the patient and his parents by using NucleoSpin tissue extraction kit (Macherey-Nagel, Düren, Germany). The panel was designed using Nextera technology on a MiSeq platform (Illumina, San Diego, CA, USA), following the manufacturer’s protocol, with expected coverage of 99% of the targeted genomic regions. Mapping of sequences against the hg19 reference genome was performed by Bowtie2. Bioinformatic tools HaplotypeCaller (GATK v.4.3) and ANNOVAR were used to call and annotate the variants, respectively. Variants were filtered so that to include only variants covered by at least 20 reads and with mapping quality values exceeding a Phred-score of 30. Variants were analyzed under presumed autosomal recessive, dominant or de novo inheritance models. Variants of the proband were filtered to retain all variants predicted to have functional impact (i.e., nonsynonymous variants and changes affecting splice sites) by available bioinformatics tools including PolyPhen-2 (http://genetics.bwh.harvard.edu/pph2/, accessed on 9 December 2019), Sorting intolerant from tolerant (http://sift.jcvi.org/, accessed on 9 December 2019), Mutation Taster (http://www.mutationtaster.org/, accessed on 9 December 2019), Alamut (http://www.interactive-biosoftware.com/, accessed on 9 December 2019) and Combined annotation dependent depletion (http://cadd.gs.washington.edu/hom, accessed on 9 December 2019). List of the rare/private (gnomAD frequency <0.5%, population-matched in-house DB frequency <1%) variants predicted to have functional impact (CADD score > 15). Segregation was verified by Sanger sequencing in the families. Accession numbers are as follows: human *KCNA2* mRNA: NM_004974.4; human *KCNA2* protein: NP_004965.1.

### 4.3. Mutagenesis and Expression of Kv1.2 WT and Mutant Channel

The E236K mutation was introduced into the plasmid pIRES-KCNA2-AcGFP1 containing the full-length WT hKv1.2 cDNA using the QuickChange™ site-directed mutagenesis kit (Stratagene Cloning Systems, Santa Clara, CA, USA). The complete coding region of the cDNA was sequenced to exclude polymerase errors. HEK293 cells were transiently transfected with the Kv1.2 WT or E236K (7 μg) and CD8 reporter plasmids (1 μg) using the calcium–phosphate precipitation method. Only cells bound with anti-CD8 antibody-coated microbeads (Dynabeads M450, ThermoFisher Scientific, Waltham, MA, USA) were used for patch-clamp recordings.

### 4.4. Electrophysiology

Standard whole-cell patch-clamp recordings were performed at room temperature (~20 °C) using an Axopatch 200B amplifier (Axon Instruments, Sunnyvale, CA, USA). The bath solution contained (mM): NaCl 142, KCl 2.8, MgCl_2_ 1, CaCl_2_ 1, HEPES 10, glucose 11, pH = 7.4 whereas the pipette solution contained (mM): NaCl 10, K-glutamate 132, MgCl_2_ 2, CaCl_2_ 0.9, EGTA 1, HEPES 10, pH = 7.4 [48]. Pipettes were pulled from borosilicate glass and had ~2.5 MΩ resistance.

Outward currents were evoked by 400 ms depolarizing commands from a holding potential of –80 mV to +40 mV in 5 mV intervals, followed by a 150 ms voltage step at −20 mV. To measure tail currents, this voltage protocol was preceded by a depolarizing pulse of 500 ms at +60 mV to favor the transition of the channel from slow to fast gating [20,49]. The voltage-dependence of channel activation was determined by plotting normalized tail currents as a function of membrane potential, and fitting data points with the Boltzmann function I = 1/1 + exp{ −(V − V_1/2_)/k}. V_1/2_, the half-maximal activation potential and k, the slope factor, were calculated from fit.

To measure steady-state inactivation, cells were depolarized to various pre-pulse potentials, from −100 to +20 mV in +10 mV increments for 4 s, and then held at +40 mV test potential for 300 ms. the voltage dependence of steady state inactivation was derived by plotting normalized peak current amplitudes at +40mV as a function of the pre-pulse potentials and fitting data points with the Boltzmann function.

To measure activating kinetics, currents were elicited by 400 ms depolarizing pulses from a holding potential of –80 mV to +40 mV in 5 mV intervals. To measure deactivating kinetics, currents were elicited by 400 ms depolarizing pulse at +20 mV followed by 200 ms depolarizing pulses from −80 to +20 mV in 5 mV intervals. Activating and deactivating kinetics were measured by fitting activating and deactivating current traces with a single exponential function. The resulting time constants were plotted as a function of voltage and fitted with the equation: τ = τV_1/2_ exp(V − V_1/2_)/k, where τV_1/2_ is the time constant at the mid-point activation voltage (V_1/2_) of the channels, and k is the slope factor for the voltage-dependence of the time constants.

To determine the C-type inactivation kinetics, a test pulse to +20 mV was delivered for 90 s to cells expressing Kv1.2 channels. The slow inactivation was estimated by fitting the time course of current decay with a single-exponential function and calculating the time constant (τ) and the relevant amplitudes (A%).

Currents were low-pass filtered at 2 kHz and digitized with sampling rates of 50 kHz using the Digidata 1440A AD/DA converter (Molecular Devices, Sunnyvale, CA, USA). Data were analyzed by using pClamp 10.3 (Molecular Devices, Sunnyvale, CA, USA) and Kaleida Graph Software.

For the pharmacological experiments, 4-AP was daily dissolved in the bath solution. To quantify the effect of 4-AP, potassium currents elicited from Kv1.2 and E236K channels were recorded before and after the application of 4-AP at concentrations from 3 μM−30 mM. IC_50_ was determined by calculating the ratio of the steady-state current in the presence I(c) and absence I(0) of the drug at different concentrations and fitting the ratios to the equation: I(c)/I(0) = 1/(1 + c/IC_50_), where c is the concentration.

Results are reported as mean ± SEM of n cells from at least three different transfection experiments. Statistical analysis was performed using Student’s *t*-test, with *p* < 0.05 or less considered as significant.

### 4.5. Homology Modeling and Molecular Dynamic Simulations

A homology model of human Kv1.2 was built from the crystal structure of the Kv1.2/2.1 chimera (PDB 2R9R) [50]. Residues that differed or were missing in the Kv1.2/2.1 chimera as compared with the human Kv1.1 were replaced and modeled, respectively, using Modeller 9.25 [51,52] We introduced the mutation E236K in two adjacent of the four subunits in the Kv1.2 tetramer. The resulting heterotetramer with two wild-type and two mutant subunits was introduced into a POPE: POPC: PSPS membrane (3:2:1) using CHARMM-GUI [53,54,55] and was equilibrated using NAMD 2.14 [56]. Figures have been generated using Pymol 2.5 (https://pymol.org/2/).

## Figures and Tables

**Table 1:** Biophysical parameters of Kv1.2 WT (7 μg), E236K (7 μg) and Kv1.2 WT+E236K (3.5 + 3.5 μg) channels expressed in HEK 293 cells.

	Current Density +40 mV/−10 mV	Voltage Dependence of Activation		Kinetic of Activation	Kinetic of Deactivation	Steady-State Inactivation	
	nA	V_1/2_ (mV)	k (mV)	τ_V1/2_ (ms)	τ_V1/2_ (ms)	V_1/2_ (mV)	k (mV)
**Kv1.2 WT**	0.7 ± 0.2 0.08 ± 0.01 (28)	−11.5 ± 0.9 (9)	11.5 ± 0.8	18.5 ± 1.05 (9)	55.5 ± 1.5 (13)	−34.0 ± 3.0 (8)	20.0 ± 3.0
**E236K**	1.0 ± 0.3 0.27 ± 0.04 (28)	−29.4 ± 1.0 * (15)	13.7 ± 1.0	96.1 ± 8.5 * (16)	101.4 ± 3.3 * (10)	−32.9 ± 1.3 (7)	18.4 ± 1.2
**Kv1.2 WT+E236K**	0.7 ± 0.9 0.20 ± 0.03 (23)	−18.8 ± 0.9 * (18)	12.0 ± 0.6	45.5 ± 0.9 * (16)	93.1 ± 2.0 * (14)	−27.1 ± 0.8 (n = 13)	16.3 ± 0.8

Data are mean ± SEM of the number of cells indicated in parenthesis. * *p* < 0.05, with respect to WT.

## Data Availability

The data that support the findings of this study are available from the corresponding authors, upon reasonable request.

## Supplementary Materials

The following are available online at https://www.mdpi.com/article/10.3390/ijms22189913/s1.
