# Supplementary material for: A Novel KCNA2 Variant in a Patient with Non-Progressive Congenital Ataxia and Epilepsy: Functional Characterization and Sensitivity to 4-Aminopyridine
Source: Int J Mol Sci. 2021 Sep 14;22(18):9913. doi: 10.3390/ijms22189913 (PMC8469797; doi:10.3390/ijms22189913)
Supplement: Supplementary file 1 [file ijms-22-09913-s001.zip › ijms-1321554-supplementary.pdf]

Supplemental materials

Table S1. Clinical-functional correlations of individuals harboring *KCNA2* variants compared to the present case. The percentage of cases with specific clinical features is stated in relation to the number of cases with available clinical and functional data. CSWS= continuous spikes and waves during slow sleep. n.d = not determined.

Functional class	Present study (GOF/LOF)	GOF/LOF	GOF	LOF
Development before seizure onset	Normal	37.5%	75%	85%
Epilepsy onset	Neonatal or early infantile (18 months)	100%	0%	0%
Seizure types	Generalized only	30%	69%	46%
Febrile seizures	Yes	10%	41%	48%
Seizure outcome	Drug responsive	0%	15%	40%
Intellectual disability	Mild (IQ=56)	10%	21%	25%
Ataxia	Yes	100%	100%	64%
Tremor	Yes	20%	44%	25%
Spasticity	No	43%	100%	75%
CSWS	No	100%	100%	55%
Brain MRI	Cerebellar atrophy	50%	57%	0.7%
Clinical course	Non progressive	n.d	n.d	n.d
